# Off-label use of rhBMP-2 as bone regeneration strategies in mandibular ameloblastoma unicystic

**DOI:** 10.1590/S1679-45082017RC3777

**Published:** 2017

**Authors:** Henrique Celestino Lima e Silva, Adonai Peixoto Cheim, Roberto Moreno, Sérgio Luis de Miranda

**Affiliations:** 1 Hospital Israelita Albert Einstein, São Paulo, SP, Brazil.

**Keywords:** Bone morphogenetic proteins, Ameloblastoma, Mandibular neoplasms, Bone regeneration, Bone transplantation, Case reports

## Abstract

Jawbone reconstruction after tumor resection is one of the most challenging clinical tasks for maxillofacial surgeons. Osteogenic, osteoinductive, osteoconductive and non-antigenic properties of autogenous bone place this bone as the gold standard for solving problems of bone availability. However, the need for a second surgical site to harvest the bone graft increases significantly both the cost and the morbidity associated with the reconstructive procedures. Bone grafting gained an important tool with the discovery of bone morphogenetic proteins in 1960. Benefit of obtaining functional and real bone matrix without need of second surgical site seems to be the great advantage of use bone morphogenetic proteins. This study analyzed the use of rhBMP-2 in unicystic ameloblastoma of the mandible, detailing its structure, mechanisms of cell signaling and biological efficacy, in addition to present possible advantages and disadvantages of clinical use of rhBMP-2 as bone regeneration strategy.

## RESUMO

A reconstrução óssea dos maxilares após ressecções tumorais é uma das tarefas mais difíceis para o cirurgião maxilofacial. As propriedades osteogênicas, osteoindutoras, osteocondutoras e não antigênicas do osso autógeno o colocam como o padrão-ouro para a solução de problemas de disponibilidade óssea. Entretanto a coleta do enxerto ósseo necessita de um segundo sítio cirúrgico, aumentando significativamente o custo e a morbidade associados ao procedimento reconstrutivo. A enxertia óssea ganhou uma excelente ferramenta com a descoberta das proteínas ósseas morfogenéticas na década de 1960. O benefício da obtenção de matriz óssea verdadeira e funcional, sem a necessidade de um segundo sítio cirúrgico, parece ser a grande vantagem do uso das proteínas ósseas morfogenéticas. Neste contexto, o objetivo deste estudo foi analisar a utilização da rhBMP-2 na regeneração óssea de ameloblastoma mandibular unicístico, detalhando sua estrutura, seus mecanismos de sinalização celular e sua eficácia biológica, além de apresentar potenciais vantagens e desvantagens da utilização clínica das rhBMP-2, enquanto estratégia regenerativa.

## INTRODUCTION

Ameloblastoma is a tumor affecting maxillary bones, and it originates from odontogenic epithelium. This is a benign tumor that arising from epithelial remaining of tooth embryogenesis.^1^ Approximately 80% of ameloblastoma cases occur in the jawbone, mainly in the ascending ramus and molar region.^1-3^

In general, amelobastomas are asymptomatic and diagnosed during routine radiographic exams.^1^ The surgery is highlighted among treatment options available for ameloblastoma, surgical extension depends on the size of the lesion.^2^

Bone reconstructions are required after surgical treatment of these injuries and, in this context, the development of bone morphogenic proteins (BMP) in 1960 constituted an excellent tool for bone grafting.^4^

A number of studies have investigated the use of BMP in different regenerative applications in many areas of medicine.^4,5^ In dentistry the rhBMP-2 is used to enhance results of regeneration in surgery of large mandibular or maxillary bone defects, and also for periodontal surgery, surgical resection associated with tumor injuries and adaptation of dental implants.^4,5^

This study analyzed main characteristics and structure of rhBMP-2 focusing on its regenerative applications in maxillofacial surgery. We report a case of a 15-year-old boy with unicystic ameloblastoma, who were treated with enucleation and curettage associated with bone reconstruction using rhBMP-2 (INFUSE Bone Graft^®^, Medtronic Sofamor Danek, Memphis, Tennessee, United States).

## CASE REPORT

A 15-year-old boy with leucoderma was admitted to our service reporting pain while chewing, bulging in the left mandibular angle and aesthetic discomfort because of face asymmetry, but no relevant clinical facts existed.

**Figure 1:**
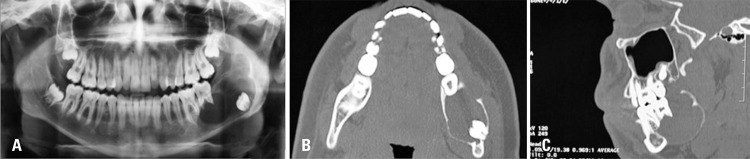
(A) Panoramic radiographic before the surgery. A radiolucent injury, unicystic pattern, unilocular, transition zones defined by thin sclerotic border, discreet insufflation extending from mandibular body to left ascending ramus, near to sigmoid notch associated with radicular reabsorption of teeth 36 and 37, and displacement tooth 38 for mandibular basis. (B) Computed tomography, axial cut. It is possible to observe a cystic injury of uniloculated aspect with homogenous and hypodense density in relation to muscle tissues with extension of body to left ascending ramus with signals of rupture of alveolar cortical and displacement of tooth 38. (C) Computed tomography, sagittal cut. A cystic injury of uniloculated aspect and radicular reabsorption of teeth 36 and 37 are also observed

Physical examination revealed a facial asymmetry, a palpable mass in the angle region of left mandibular, pain, limitation of oral opening and bulging in region of teeth 36 and 37, tooth 38 was lacking. The diagnosis exam, the panoramic radiographic and computed tomography of the face showed suggestive images of injury involving the body and left mandibular ascending ramus (Figures 1A to C).

**Figure 2:**
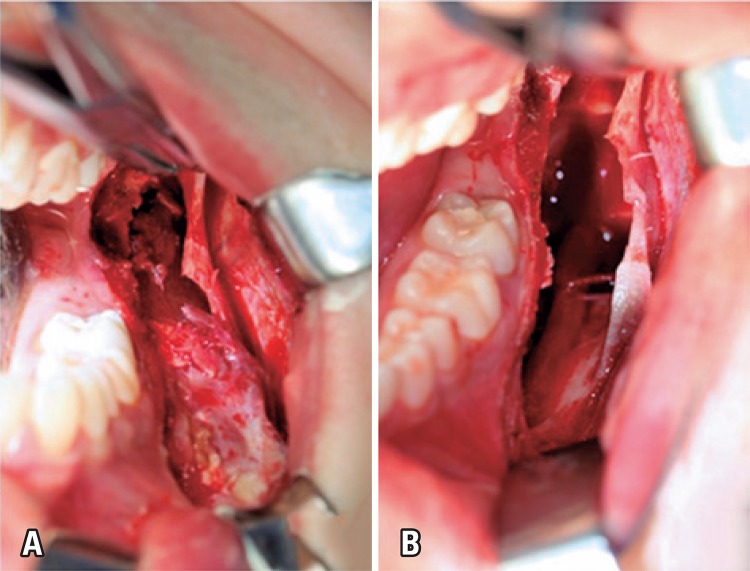
(A) Aspect of the injury in the surgery. Intraoral access of surgical removal of the lesion. (B) enucleation and curettage of the lesion with simple dissection because the tumor was encapsulated, , well-defined limits and cleavage plan, characteristics that enable its complete surgical removal

Patient’s familiar medical history did not show significant data. Reconstruction possibilities were discussed with patient and his family including the use of autogenous bone grafting of iliac crest and/or off-label use of rhBMP-2. Advantages and disadvantages of both possibilities were presented and patient and family decided for rhBMP-2 off-label. After evaluation of surgical risk and treatment of teeth 36 and 37 canals had are done, the patient underwent surgery in a hospital environment under general anesthesia and nasotracheal intubation. We performed an incision with extension of mandibular ascending ramus to vestibular region to first lower left molar, being elevated the mucoperiosteal graft, therefore achieving a partial exposition of the injury. An osteotomy was need using a 701 drill for higher exposure of the injury (Figure 2A). The dissection was simple because the tumor was encapsulated (Figure 2B).

**Figure 3:**
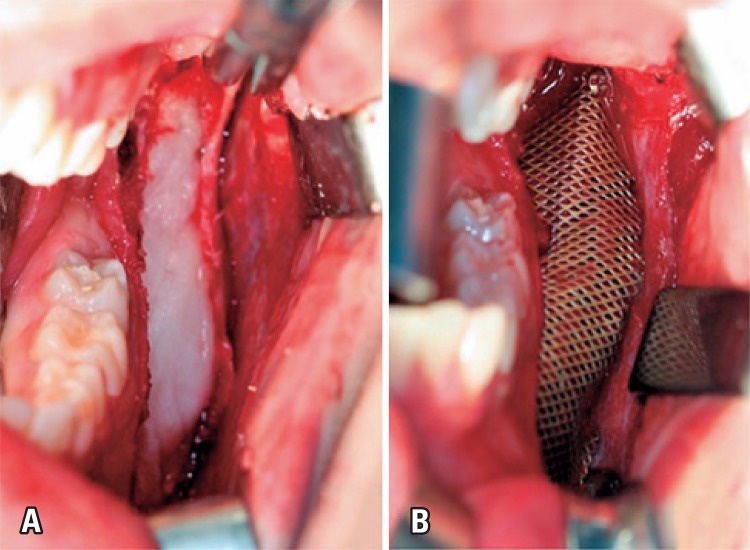
(A) Prepared recipient site. The bone grafting technique defined with the use of rhBMP-2. Adaptation of titanium mesh 1.0 system

Recipient site was prepared and, by using the bone grafting technique, the rhBMP-2 was prepared associated with Bio-Oss^®^ (Geistlich Pharma AG, Wolhusen, Switzerland) and fairly adapted to surgical site with subsequent adaptation of titanium mesh 1.0 system that promoted a mechanical barrier. A plate 2.0 system was used on external oblique line to obtain higher stability (Figures 3A and B).

**Figure 4:**
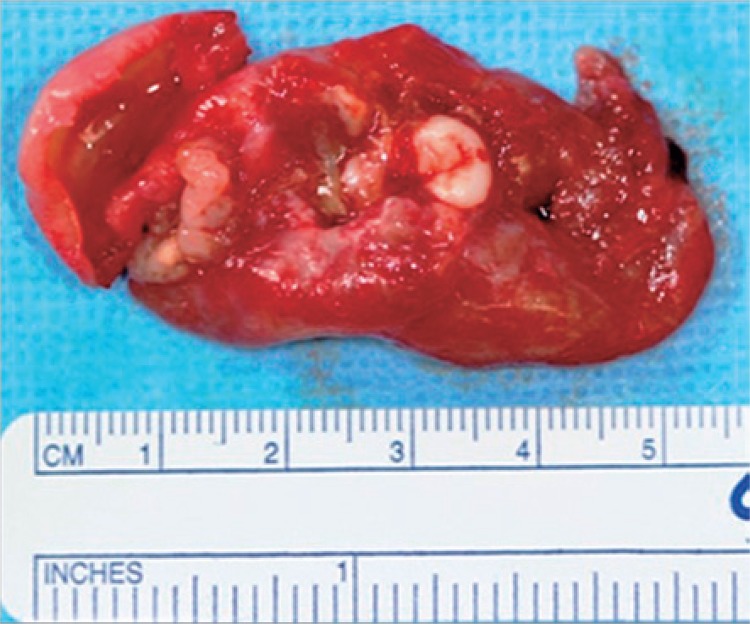
Macroscopic aspect of the lesion

A polyglactin 910 wire suture was used in deep plans and on the mucosa. Material obtained was forwarded for anatomopathological examination, and the definitive diagnosis was unicystic ameloblastoma, intraluminal variant with plexiform pattern (Figure 4).

**Figure 5:**
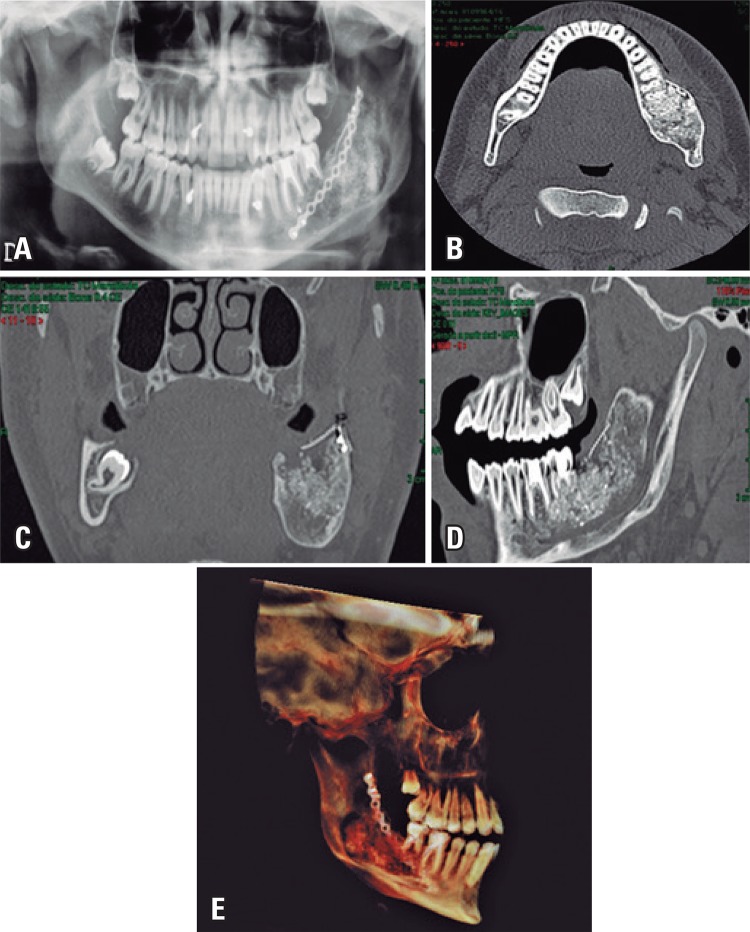
(A) Panoramic radiography. Recipient site can be observed with satisfactory adaptation of rhBMP-2 associated with Bio-Oss®. (B) Computed tomography. Axial cut is shown with satisfactory adaptation of grafting material in the recipient site. (C) Computed tomography. Coronal cohort is observed, showing satisfactory adaptation of grafting material and recipient area fixation. (D) Computed tomography. Observe a sagittal section, evidencing satisfactory adaptation of the grafting material and the titanium mesh in the receiving area. (E) Computed tomography. Tri-dimensional reconstruction for follow-up after the surgery

Immediately after surgery, the patient had an edema compatible with the procedure and discreet paresis of left inferior alveolar nerve. We observed a late inflammation reaction. Clinical and radiographic follow-up was done after 1, 3 and 6 months with satisfactory aesthetic and functional result (Figures 5A and E).

## DISCUSSION

The first reports in the literature on the mandibular reconstruction technique obtained success for treatment of bone defects of 6cm after enucleation and curettage of unicystic ameloblastoma with association of rhBMP-2. These studies observed clinical and radiographic evidences of new bone formation after 3, 6 and 9 months, and they did not mentioned complications after the surgery. However, in a later control for 9 months, these reports choose to analyze histologically the new bone format that characteristics mimic native bones.^6^ In 2007, a study that used rhBMP-2 reported that formation of new bone is dose-dependent,^7^ and it also reported the possible occurrence of exaggerated bone growth, edema and reabsorption of native bone, in case that indicated dose is not respected or if the ability of the carrier would be exceeded.

Reconstruction of large mandibular defects with rhBMP-2 off-label was described in 2008.^8^ Defects of all patients had resolved and, after 6 months, all injuries were replaced by mineralized bone such as the one use in our study. Another study from 2008 reported five cases whose patients needed mandibular bone reconstruction after resection of unicystic ameloblastoma. Reconstruction was done using rhBMP-2 with concentration of 1.5mg/mL in collagen sponge. Of five cases, three had success. The fail of treatment in these two cases is explained by the chronic infection and by the lack of maintenance of grafting space.^9^

A literature review analyzed all available papers on reconstructions of bone defects with critical sizes published until 2011 and reported that from 37 patients treated with rhBMP-2, 86.5% of them had success and 13.5% failed in adequate formation of bone. In these studies, the reconstruction of bone defects with this technique is viable and, in general, reconstruction with rhBMP-2 were well-tolerated, even in cases with significant edema after surgery,^10^ similar to the case described in our study.

## References

[1] Neville BW, Allen CM, Bouquot JE (2004). Patologia oral & maxilofacial.

[2] Regezi JA, Sciubba JJ (2000). Patologia bucal: correlações clinicopatológicas.

[3] Hatada K, Noma H, Katakura A, Yama M, Takano M, Ide Y (2001). Clinicostatistical study of ameloblastoma treatment. Bull Tokio Dent Coll.

[4] Bragdon B, Moseychuk O, Saldanha S, King D, Julian J, Nohe A (2011). Bone morphogenetic proteins: a critical review. Cell Signal.

[5] Xiao YT, Xiang LX, Shao JZ (2007). Bone morphogenetic protein. Biochem Biophy Res Commun.

[6] Moghadam HG, Urist MR, Sandor GK, Clokie CM (2001). Successful mandibular reconstruction using a BMP bioimplant. J Craniofac Surg.

[7] Solofomalala GD, Guery M, Lesiourd A, Le Huec JC, Chauveaux D, Laffenetre O (2007). Bone morphogenetic proteins: from their discoveries till their clinical applications. Eur J Orthop Surg Traumatol.

[8] Herford AS, Boyne PJ (2008). Reconstruction of mandibular continuity defects with bone morphogenetic protein-2 (rhBMP-2). J Oral Maxillofac Surg.

[9] Carter TG, Brar PS, Tolas A, Beirne OR (2008). Off-label use of recombinant human bone morphogenetic protein-2 (rhBMP-2) for reconstruction of mandibular bone defects in humans. J Oral Maxillofac Surg.

[10] Herford AS, Stoffella E, Tandon R (2011). Reconstruction of mandibular defects using bone morphogenic protein: can growth factors replace the need for autologous bone grafts? A systematic review of the literature. Plast Surg Int.

